# Exomic Sequencing of Four Rare Central Nervous System Tumor Types

**DOI:** 10.18632/oncotarget.964

**Published:** 2013-04-06

**Authors:** Chetan Bettegowda, Nishant Agrawal, Yuchen Jiao, Yuxuan Wang, Laura D. Wood, Fausto J. Rodriguez, Ralph H. Hruban, Gary L. Gallia, Zev A. Binder, Callen J. Riggins, Vafi Salmasi, Gregory J. Riggins, Zachary J. Reitman, Ahmed Rasheed, Stephen Keir, Sueli Shinjo, Suely Marie, Roger McLendon, George Jallo, Bert Vogelstein, Darell Bigner, Hai Yan, Kenneth W. Kinzler, Nickolas Papadopoulos

**Affiliations:** ^1^ Department of Neurosurgery, Johns Hopkins University School of Medicine, Baltimore, MD, USA; ^2^ Ludwig Center for Cancer Genetics, Johns Hopkins University School of Medicine, Baltimore, MD, USA; ^3^ Department of Otolaryngology-Head and Neck Surgery, Johns Hopkins University School of Medicine, Baltimore, MD, USA; ^4^ Department of Pathology, Johns Hopkins University School of Medicine, Baltimore, MD USA; ^5^ Department of Anesthesiology, Cleveland Clinic Hospital, Cleveland, OH USA; ^6^ Preston Robert Tisch Brain Tumor Center, Duke University Medical Center, Durham, NC, USA; ^7^ Department of Pathology, University of São Paulo School of Medicine, São Paulo, Brazil

**Keywords:** Central nervous system (CNS) tumors, cancer genetics, exome sequencing, pediatric tumors, brain tumors

## Abstract

A heterogeneous population of uncommon neoplasms of the central nervous system (CNS) cause significant morbidity and mortality. To explore their genetic origins, we sequenced the exomes of 12 pleomorphic xanthoastrocytomas (PXA), 17 non-brainstem pediatric glioblastomas (PGBM), 8 intracranial ependymomas (IEP) and 8 spinal cord ependymomas (SCEP). Analysis of the mutational spectra revealed that the predominant single base pair substitution was a C:G>T:A transition in each of the four tumor types. Our data confirm the critical roles of several known driver genes within CNS neoplasms, including *TP53* and *ATRX* in PGBM, and *NF2* in SCEPs. Additionally, we show that activating *BRAF* mutations play a central role in both low and high grade glial tumors. Furthermore, alterations in genes coding for members of the mammalian target of rapamycin (mTOR) pathway were observed in 33% of PXA. Our study supports the hypothesis that pathologically similar tumors arising in different age groups and from different compartments may represent distinct disease processes with varied genetic composition.

## INTRODUCTION

The annual incidence of brain and other nervous system tumors in the United States is approximately 23,000, with an associated annual mortality of nearly 14,000[1]. These tumors comprise a widely heterogeneous array of neoplasms, each affecting an extremely small but important fraction of the population. Given their rarity, these tumors are difficult to study and clinical decision-making is often based on extrapolated data generated from adult tumors with similar pathological and histological findings. However, it is becoming increasingly apparent that pediatric and adult tumors are widely disparate in their biology, genetics, and clinical behavior. Much work has already been conducted on the global genetic profiling of adult glioblastomas and pediatric medulloblastoma[2-9]. Pontine gliomas and anaplastic oligodendrogliomas have also been sequenced at a genome-wide level[10-13]. In this work, we studied four tumors, with either astrocytic or ependymal differentiation: 1) pleomorphic xanthoastrocytoma (PXA), 2) non-brainstem pediatric glioblastoma (PGBM), 3) intracranial ependymoma (IEP) and 4) spinal cord ependymoma (SCEP).

PXAs (Figure 1A) are rare astrocytic malignancies that are classified by the World Health Organization (WHO) as grade II lesions. They typically present with seizures and are often superficially located within the brain. The current treatment paradigm calls for maximal safe surgical resection, with no clear role for adjuvant radiation or chemotherapy[14]. The overall prognosis is favorable, with a ten-year survival rate of 70%[15]. However, malignant transformation has been reported in nearly a third of these tumors[15, 16].

**Figure 1A:**
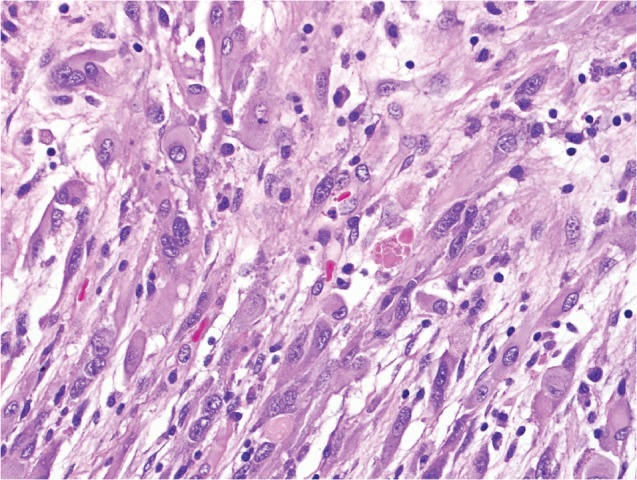
Pleomorphic xanthoastrocytoma (WHO grade II). Pleomorphic xanthoastrocytoma represents a distinctive glioma subtype. Histologically it is characterized by the presence of nuclear pleomorphism (e.g. variation in nuclear size), a fascicular arrangement of cells and eosinophilic granular bodies (arrow).

Grade IV astrocytomas (Figure 1B), otherwise known as glioblastoma, are uniformly lethal tumors with a two-year life expectancy of only 5-30% in children[17, 18]. PGBMs are amongst the most challenging pediatric tumors to treat and do not respond to even the most aggressive regimens, including surgery, radiation, and chemotherapy[19]. PGBMs are typically classified as either arising from the brainstem (diffuse pontine glioma) or non-brainstem glioblastoma. The current study focuses on non-brainstem lesions.

**Figure 1B:**
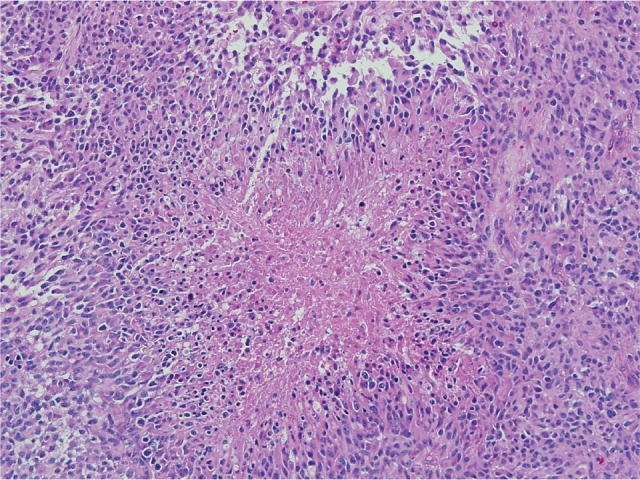
Pediatric glioblastoma (WHO grade IV). Morphologic features of pediatric glioblastomas are similar to those found in adult patients. Pseudopalisading necrosis is a characteristic finding (center), and diagnostic of glioblastoma when present in a mitotically active infiltrating astrocytoma.

Ependymoma is the third most common pediatric brain tumor and these neoplasms exhibit differentiation in the direction of cells lining the ventricular system in the brain and the central canal in the spinal cord. Over 90% of pediatric ependymomas are intracranial, while the vast majority of adult ependymomas arise in the spinal cord[20]. Intracranial ependymomas (IEPs) (Figure 1C) are challenging lesions to treat, with surgery being the mainstay of therapy given the lack of efficacious chemotherapy or radiation regimens. Progression free-survival is only 30% at 5 years, with 5 year-survival rates of 60%[20]. Spinal cord ependymomas (SCEPs) (Figure 1D) are rare neoplasms that are typically WHO grade I- III lesions that can be cured with gross total resection. However, given the delicate nature of the spinal cord, post-operative neurological deficits are common. In addition, when complete surgical excision is not possible, chemotherapeutic strategies have not been shown to be of benefit. Given the paucity of effective adjuvant therapy, 5 year-survival rate for grade III SCEPs is ~65%[21].

**Figure 1C:**
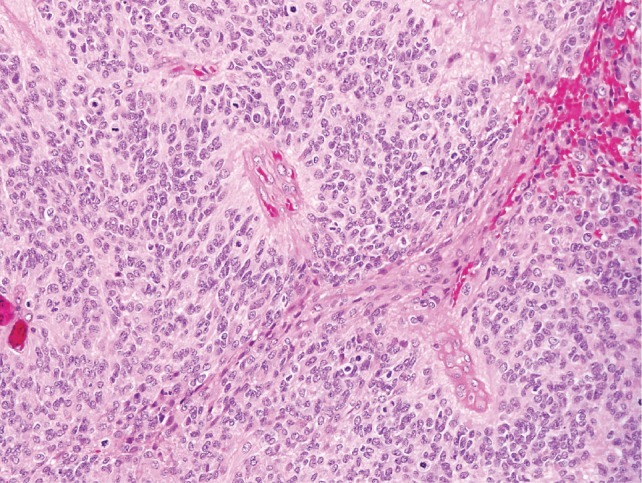
Intracranial ependymoma (WHO grade III). Perivascular pseudorosettes are a histologic hallmark of ependymoma, and represent neoplastic cell processes surrounding intratumoral vessels. Brisk mitotic activity is present in this intracranial ependymoma (arrows), which suffices to classify it as anaplastic.

**Figure 1D:**
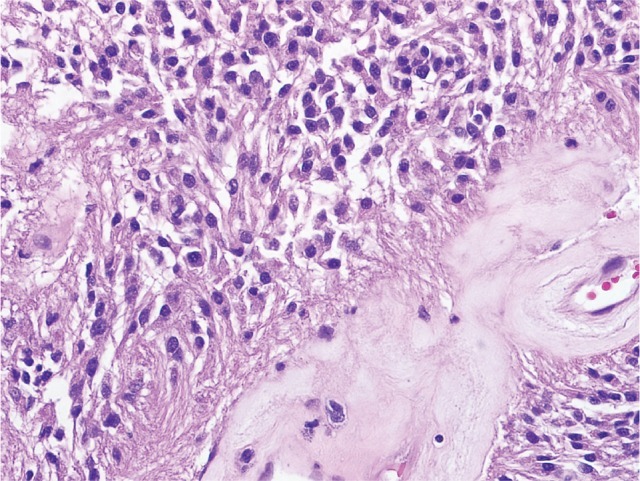
Spinal cord ependymoma (WHO grade II). Spinal cord ependymomas are typically low grade, and demonstrate low proliferative activity. Perivascular pseudorosettes, composed of numerous perivascular glial processes (arrows), are frequent characteristic histologic features.

To understand better the pathogenesis of astrocytic and ependymal tumors, we sequenced the coding exons of 20,687 genes in tumor and matched normal DNA from 12 PXA, 17 non-brainstem PGBM, 8 IEP and 8 SCE using massively-parallel sequencing. Using a previously described process, all sequencing data were passed through a series of stringent filters[12, 22, 23]. All putative alterations were then manually inspected and curated and a subset of mutations was validated using Sanger sequencing.

## RESULTS

We evaluated 12 PXAs from ten patients; in two patients, we were able to analyze both the primary and recurrent tumors. An average of 6.7 × 10^9^ high quality bases were sequenced per sample, with an average of 72 distinct reads per targeted base; 90% of these bases having at least 10 distinct reads (Table 1). After filtering and visual inspection, the sequencing results revealed 113 high confidence somatic mutations in the 12 samples (see Materials and Methods). We attempted to confirm a subset of mutations found in this study using independent platforms, such as Sanger Sequencing. As expected from our algorithms for high confidence mutations from our previous studies, greater than 90% of mutations tested were confirmed as true somatic alterations. Only seven genes were mutated in more than one of the ten primary tumors: *BRAF* was mutated in 6 tumors and *TP53*, *BAP1*, *CHERP*, *FLRT2*, and *RNF43* were each mutated in 2 tumors (Supplementary Table 1a). The tumors contained an average of 9.5 ± 8.5 mutations (range 1 to 28). In the recurrent case (XPA25PT3) that histopathologically progressed, there was a *de novo* truncating mutation in *RB1*, as well as a mutation in *PIK3R1* that was not detected in the original PXA (Supplementary Table 1a). That tumor also had far higher levels of LOH than the other cases at the time of initial resection, at which point it was still pathologically defined as a PXA (Table 2). Genes coding for members of the mTOR pathway were altered in 4/12 of PXA samples, *NF1* alterations in 2 cases, *TSC2* and *PIK3R1* in 1 case each. No mTOR pathway gene alterations were identified in cases with *BRAF* mutation.

**Table 1:** Summary of Sequence Analysis

	PXA		PGBM		IEP		SCEP	
Coverage Summary	Tumor	Normal	Tumor	Normal	Tumor	Normal	Tumor	Normal
Number of samples analyzed	12	12	17	17	8	8	8	8
Bases sequenced (after quality filtering)	6.7 ± 3.9 × 10^9	8.4 ± 1.4 × 10^9	13.1 ± 4.0 × 10^9	12.6± 3.5 × 10^9	9.3 ± 0.49 × 10^9	9.88 ± 1.6 × 10^9	7.1 ± 1.1 × 10^9	7.2 ± 1.2 × 10^9
Bases mapped to targeted region	3.2 ± 1.5 × 10^9	3.8 ± 0.69 × 10^9	6.6 ± 2.2 × 10^9	6.8 ± 2.0 × 10^9	4.5 ± 0.29 × 10^9	4.7 ± 083 × 10^9	3.6 ± 0.54 × 10^9	3.7 ± 0.64 × 10^9
Average # of distinct reads per targeted base	72.4± 33.3	93 ±15.6	108.0 ± 36.1	119.0 ± 37.5	101.2 ± 6.2	107.3 ± 10.6	80.6 ± 10.2	87.9 ± 14.8
Targeted bases with at least 10 distinct reads (%)	90 ± 11%	95 ± 2.7%	94 ± 1.5%	94.6 ± 1.05%	96.8 ± 0.2%	96.3 ±0.48%	77 ± 2.5%	96.2 ±0.59%
**Tumor and normal comparison**								
Known SNPs identified in tumor	15,466 ± 2816		22,126 ± 2209		17,464 ± 1885		11,342 ± 646	
% tumor SNPs identified in matched normal	99.9± 0.01%		99.5± 0.01%		99.9± 0.02%		99.9± 0.02%	
Non-synonymous somatic mutations in tumor	9.6± 8.3		23.5 ± 11.2		12.8 ± 10.6		12.9 ±6.4	

**Table 2:** LOH Heat Map

PXA																								
Chromosome	1	2	3	4	5	6	7	8	9	10	11	12	13	14	15	16	17	18	19	20	21	22	X	Y
**Sample:**																								
XPA21PT																								
XPA23PT																								
XPA24PT																								
XPA25PT1																								
XPA25PT3																								
XPA26PT																								
XPA27PT1																								
XPA27PT2																								
XPA28PT																								
XPA31PT																								
XPA33PT																								
XPA34PT																								

PGBM																								
**Sample:**																								
PGBM01PT2																								
PGBM02PT																								
PGBM03PT																								
PGBM04PT																								
PGBM05PT																								
PGBM07PT																								
PGBM09PT																								
PGBM10PT																								
PGBM11PT																								
PGBM13PT																								
PGBM15PT																								
PGBM16PT																								
PGBM17PT																								
PGBM18PT																								
PGBM20PT																								
PGBM32PT																								
PGBM33PT																								

Intracranial Ependymomas																								
**Sample:**																								
SE16PT																								
SE18PT																								
SE20PT																								
SE21PT																								
SE22PT																								
SE30PT																								
SE31PT																								
SE35PT																								

Spinal Ependymomas																								
**Sample:**																								
SE1PT																								
SE3PT																								
SE4PT																								
SE5PT																								
SE6PT																								
SE7PT																								
SE8PT																								
SE9PT																								
	No LOH																							
	Partial LOH																							
	Complete LOH																							

Exomic sequencing and analysis was performed on 17 PGBMs, generating an average of 13.1 × 10^9^ high quality bases per sample. A mean of 108 distinct reads per targeted base was achieved, with 94% of targeted bases represented by at least 10 distinct reads (Table 1). The PGBMs contained an average of 23.5 ± 11.2 mutations (range 4-46). The recurrently mutated genes in our cohort of PGBMs were *TP53* (7/17), *ATRX* (3/17), *TTN* (3/17), *BRAF* (2/17), *IDH1* (2/17), *FRMPD4* (2/17), *PKHD1L1* (2/17), *PTPRU* (2/17), *SPATA22* (2/17) (Supplementary Table 1b). Of note, recently described alterations in the histone *H3.3* gene was detected in only 1 of 17 samples via next generation sequencing[10, 11]. *H3.3* and *BRAF* were subsequently evaluated by Sanger Sequencing in the same samples and six additional PGBMs. In all, *H3.3* mutations were identified in two of the 23 tumors, while *BRAF* mutations were identified in 3 of 23 tumors.

Sequencing the coding regions of 8 IEPs generated 9.3X10^9^ high quality bases with an average of 101 distinct reads and 97% with at least 10 distinct reads. For the 8 SCEPs analyzed, 7.1X10^9^ high quality bases were generated, with an average of 81 distinct reads per targeted base and 77% of bases with at least 10 distinct reads (Table 1). The IEPs contained an average of 12.8 ± 10.6 mutations (range 5 to 34). Notably, there were no recurrent mutations in the IEPs (Supplementary Table 1c). The only recurrent mutation in SCEPs was *NF2* (4/8), a tumor suppressor previously known to be altered in this tumor type (Supplementary Table 1d). In addition, all 8 SCEP discovery samples contained LOH of chromosome 22, where *NF2* locus resides (Table 2). The SCEPs contained an average of 12.9 ± 6.4 mutations (range 2 to 23) (Table 1). *NF2* was sequenced in an additional 48 samples comprised of 32 intracranial,11 spinal ependymomas, 3 myxopapillary ependymomas and 2 spinal sub-ependymomas. It was mutated in 5 additional samples, all of which were spinal ependymomas. In total *NF2* was mutated in 9 of 19 SCEPs (47%) and in none of 40 IEPs (0%; p<0.0002, Students t-test). However, 5/8 IEP discovery samples exhibited LOH of chromosome 22 but none contained mutations in the NF2 gene (Table 2). This suggests that another mechanism outside of NF2 may be driving loss of chromosome 22 in IEPs.

The mutational spectrum for each of the 4 tumor types was determined and in all tumors, the C:G>T:A transition was the most common single base pair alteration (Table 3). This has been shown to be the case in other CNS tumors, including medulloblastoma, adult glioblastoma and oligodendroglioma and non-CNS tumors [2, 4, 11, 12, 23-26].

**Table 3:** Mutation Spectrum

	PXA		PGBM		IEP		SCEP	
Mutations	Number	Percent (%)	Number	Percent (%)	Number	Percent (%)	Number	Percent (%)
A:T>C:G	5	4.3	26	6.5	7	6.9	13	12.6
A:T>G:C	15	13.0	54	13.5	11	10.8	17	16.5
A:T>T:A	13	11.3	36	9.0	9	8.8	14	13.6
C:G>A:T	17	14.8	55	13.8	13	12.7	14	13.6
C:G>G:C	9	7.8	44	11.0	14	13.7	9	8.7
C:G>T:A	45	39.1	140	35.0	35	34.3	29	28.2
Indel	11	9.6	45	11.3	13	12.7	7	6.8
Total	115	100.0	400	100.0	102	100.0	103	100.0
Average Number of Mutations Per Tumor	9.6		23.5		12.8		12.9	

## DISCUSSION

Our data confirm the critical roles of several known driver genes in CNS neoplasms, including *TP53* and *ATRX* in PGBM, and *NF2* in SCEPs. One particular pathway that appears to be of increasingly recognized importance is the EGFR-MAPK pathway. In adults, it has been well appreciated that alterations of *EGFR* occur in a large fraction of glioblastomas[27, 28]. Recent work also suggests that this pathway may be dysregulated in adult oligodendrogliomas, the second most common adult malignant brain tumor[12]. In pediatric brain tumors, *EGFR* alterations are infrequent but our work and that of others suggests that activating *BRAF* mutations play a central role in both low and high grade glial tumors. Alterations in *BRAF* have been previously identified in both pediatric low and high grade astrocytomas. For example, cerebellar pilocytic astrocytomas commonly harbor KIAA1549:*BRAF* fusions[29], while V600E alterations have been reported in grade II-IV astrocytomas of childhood[30-32]. Here we describe the canonical V600E *BRAF* mutation in 60% of grade II PXA cases as well as in 13% of PGBM cases. While *BRAF* mutations have been previously evaluated in PXA[33], we here demonstrate that the prevalence of other alterations in the EGFR-MAPK pathway is rare. Interestingly, none of the 16 ependymoma samples contained alterations in the EGFR-MAPK pathway; this is consistent with the idea that tumorigenesis is driven by entirely different mechanisms in different cell types within the developing central nervous system. While there are ongoing clinical trials evaluating the efficacy of BRAF inhibitors in pediatric low grade astrocytomas, perhaps it is now worth screening all pediatric astrocytic tumors, including PXA and PGBM, for MAPK alterations in the hopes of enrolling patients with a tumor with one of these mutations in rationally designed therapeutic trials. In addition, one PXA sample had only one *bona fide* somatic alteration and that was in the *BRAF* gene. This suggest that PXAs in general do not have the complex genetic background of other BRAF driven tumors, such as melanoma, and may make them particularly susceptible to BRAF inhibition. Of note, the PXA sample that had progressed was shown to acquire mutations in *RB1* and *PIK3R1*, both of which are known genetic drivers in GBM[34]. Unlike the other PXA samples, this tumor also contained an extremely high level of LOH even at the time of diagnosis of PXA, suggesting that this tumor was already more complex than most PXAs we studied.

Genes coding for members of the mTOR pathway were altered in 33% (4/12) of PXA samples. Ten of twelve PXA samples had an alteration in either *BRAF* or the mTOR pathway, in a mutually exclusive fashion, consistent with the idea that alterations of either one of these signaling cascades is required for tumorigenesis. The study of more of these rare tumors will be required to substantiate this suggestion. Recent data indicates that tumors with even infrequent alterations in the mTOR pathway can be sensitive to targeted inhibitors such as everolimus[35]. Further studies are required to investigate the utility of mTOR pathway inhibitors in the treatment of PXA, though it has proven efficacious in the treatment of sub-ependymal giant cell astrocytomas, another rare pediatric brain tumor[36].

Intriguingly, the rate of H3.3 mutations in PGBM was 2/23 (9%) in our cohort of non-brainstem glioblastoma, a frequency much lower than the 22-31% reported in recent publications[10, 11]. Some as yet unappreciated difference in the patients evaluated in our study compared to those evaluated in the other studies must exist. Of note, one of the two PGBM cases that harbored an alteration (K27M) in H3.3 arose from the cervical spinal cord. Given that brainstem PGBMs more commonly display mutations resulting in an amino acid substitution at K27, while supratentorial PGBMs display alterations at G34, it is possible that lesions arising from the spine more closely resemble those originating from the brainstem[10, 11, 32].

Our study supports the notion that pathologically identical tumors arising from different compartments of the CNS represent divergent disease processes with varied genetic makeup and differing clinical outcomes. The genetic heterogeneity based on tumor location was most apparent with the ependymoma samples (Table 4). For example, mutations of the *NF2* gene were found exclusively in the SCEPs (9/19 samples) and never in the 40 IEPs studied (p< 0.0002; Student's t-test). There were no recurrent mutations in IEPs, despite all 8 being grade 3 (anaplastic) ependymomas and *NF2* was the only recurrent mutation in SCEPs. Overall, a very low frequency of mutations in both intracranial and spinal ependymomas was observed. One possible explanation for the lack of recurrent mutations is that the IEPs used in this study originated from different compartments of the brain. Of the 8 samples, 2 originated in the parietal lobe, 2 in the fourth ventricle, 1 in the third ventricle, 1 in the temporal lobe, 1 in the cerebello-pontine angle and 1 in the posterior fossa. Others have demonstrated that the expression profiles of ependymomas from the supratentorial compartment are different from those originating in the posterior fossa[37] and that even within the posterior fossa, there are at least two distinct molecular subtypes[38]. Ependymomas, may in fact represent a very heterogeneous class of tumors, each with distinct molecular profiles, as has been shown to be the case with PGBMs[32].

**Table 4:** Patient Demographics

PXA				
Sample:	Age:	# of Mutations:	Location:	Mutations in Recognized Driver Genes:
XPA21PT	10	12	Right temporoparietal	BRAF, TP53
XPA23PT	10	6	Not available	BRAF
XPA24PT	18	11	Not available	BRAF
XPA25PT1	11	4	Not available	TSC2
XPA25PT3	12	10	Not available	RB1, PIK3R1
XPA26PT	18	4	Not available	
XPA27PT1	18	23	Not available	NF1, TP53
XPA27PT2	18	28	Not available	NF1, TP53
XPA28PT	13	2	Not available	BRAF
XPA31PT	11	8	Left temporal	BRAF
XPA33PT	11	6	Left temporal	
XPA34PT	26	1	Right parieto-occipital	BRAF
**PGBM**				
**Sample:**	**Age:**	**# of Mutations:**	**Location:**	**Mutations in Recognized Driver Genes:**
PGBM01PT2	4	23	Left temporal/parietal/occipital	BRCA2 (has germline and somatic mutation)
PGBM02PT	20	28	Right frontal	TP53, IDH1, ATRX
PGBM03PT	12	19	Left occipital	TP53, EGFR
PGBM04PT	11	17	Posterior fossa	
PGBM05PT	9	13	Left temporal/parietal	TP53, ATRX
PGBM07PT	17	16	Right temporal	TP53, NF1
PGBM09PT	17	41	Right temporal/parietal	BRAF, ATRX
PGBM10PT	8	14	Left frontal	
PGBM11PT	7	46	Posterior fossa	PIK3C2G
PGBM13PT	2	9	Not available	
PGBM15PT	5	30	Not available	TP53
PGBM16PT	13	32	Not available	MET
PGBM17PT	18	27	Not available	
PGBM18PT	20	33	Not available	TP53, IDH1
PGBM20PT	18	28	Right temporal	BRAF
PGBM32PT	11	4	Not available	RB1
PGBM33PT	12	20	Cervical spinal cord	H3F3A, TP53
**Intracranial Ependymomas**				
**Sample:**	**Age:**	**# of Mutations:**	**Location:**	**Mutations in Recognized Driver Genes:**
SE16PT	8	5	Right parietal	
SE18PT	2	7	Fourth ventricle	
SE20PT	39	14	Posterior fossa	
SE21PT	0.9	23	Left CP Angle	
SE22PT	2	5	Right parietal	
SE30PT	52	34	Left temporal	PTEN, TP53
SE31PT	7	9	Third ventricle	
SE35PT	35	5	Fourth ventricle	HIST1H3C
**Spinal Ependymomas**				
**Sample:**	**Age:**	**# of Mutations:**	**Location:**	**Mutations in Recognized Driver Genes:**
SE1PT	43	18	T2-3	NF2
SE3PT	38	16	C4-6	
SE4PT	44	9	T3-4	
SE5PT	20	2	C3-5	
SE6PT	45	10	T3-4	NF2
SE7PT	33	11	C3-5	NF2
SE8PT	69	14	C2-4	NF2
SE9PT	77	23	T6-T7	

Developmentally, both astrocytes and ependymal cells originate from the ectoderm. However, the genetic profiles of PGBM and PXA are very different from that of IEPs and SCEPs. There were no shared driver mutations between the two classes of tumors. This likely indicates divergent inciting and pathogenic events that transform the respective cells of origin. In addition, it is possible that each of these tumors arise from different progenitor cells despite pathological and anatomic similarities.

The global genetic profiles of several malignancies, both adult and pediatric, have been published over the last five years. A few striking observations can be readily made from these and the current study: 1) the total number of mutations per pediatric tumor tends to be far fewer than adult malignancies; 2) even within childhood tumors, younger children tend to have fewer somatic alterations; 3) the number of recurrent mutations within a given tumor type is substantially less in childhood brain tumors than in adult tumors; 4) the great majority of the genes recurrently mutated in different tumor types are already well-studied, suggesting that there are a limited number of driver genes; 5) different core genes and pathways are altered in different types of pediatric malignancies; and 6) the most frequent single base substitution was a transition in all four tumor types analyzed.

As an example of these principles, we noted that the average number of mutations per PXA and IEP was 10 and 13, respectively, and for PGBM it was 25. In comparison, we found the average number of alterations in adult glioblastoma and anaplastic oligodendroglioma was 47 and 32, respectively[12, 24]. Even in PGBM, the most lethal childhood malignancy, there were samples with as few as 4 *bona fide* alterations. The overall number of mutations found in our PGBMs discovery set was comparable to what is reported in the literature. Furthermore, in our IEP discovery panel, there were three cases from adults and five pediatric cases. The average number of alterations per tumor in the adult cases was eighteen, while in the pediatric cases it was only ten.

In addition to the absolute number of mutations, the number of recurrent mutations was substantially lower in pediatric cancers. For example, in IEPs no genes were mutated in more than one sample, while in adult malignancies it is common to have a dozen or more genes recurrently mutated. Furthermore, the global genetic landscapes of pediatric and adult tumors appear to be fairly divergent. While certain genes/pathways such as *TP53* are commonly mutated in both adult and pediatric glioblastomas, only two of the pediatric tumors contained a change in *IDH1* and none harbored alterations in *IDH2*, *PTEN* or *EGFR*. Conversely, PGBMs had a much higher frequency of *ATRX* mutations when compared to primary adult glioblastoma.

Despite the heterogeneity of the genetic makeup of the four tumor subtypes analyzed, the mutational spectra were quite similar. Of note, the most common single base pair substitutions in all 4 tumor types were C:G>T:A transitions. This is a phenomenon observed in multiple CNS and non-CNS tumors and suggests susceptibility in the genome to these types of alterations.

In composite, our data suggest that CNS tumors, even those that are pathologically identical, have different molecular mechanisms that underlie their pathogenesis depending on the age of the individual and the cellular compartment from which the malignancy arises. Given the rarity of pediatric brain tumors, many clinicians are forced to draw conclusions about management paradigms based on experience and data generated from analogous adult tumors or from similar tumors arising in different parts of the central nervous system. Our findings highlight the potential perils of this approach.

## METHODS

### Preparation of tumor and matched normal samples

Fresh-frozen surgically resected tumor and matched blood were obtained from patients under an Institutional Review Board (IRB)-approved protocol from Johns Hopkins Hospital, Duke University, or University of São Paulo School of Medicine. The diagnosis of each specimen underwent central pathological review and was verified by an independent group of pathologists. Tumor tissue was analyzed by frozen section to assess neoplastic cellularity. Tumors were macrodissected to remove residual normal tissue and enhance neoplastic cellularity, as confirmed by serial frozen sections. An estimated average of 80% neoplastic cellularity was obtained.

### Preparation of Genomic DNA libraries

Genomic DNA libraries were prepared following Illumina's (Illumina, San Diego, CA) suggested protocol with the following modifications. (1) 3 micrograms (μg) of genomic DNA from tumor or normal cells in 100 microliters (μl) of TE was fragmented in a Covaris sonicator (Covaris, Woburn, MA) to a size of 100-500 bp. DNA was purified with a PCR purification kit (Cat # 28104, Qiagen, Valencia, CA) and eluted in 35 μl of elution buffer included in the kit. (2) Purified, fragmented DNA was mixed with 40 μl of H_2_O, 10 μl of 10 × T4 ligase buffer with 10 mM ATP, 4 μl of 10 mM dNTP, 5 μl of T4 DNA polymerase, 1 μl of Klenow Polymerase, and 5 μl of T4 polynucleotide kinase. All reagents used for this step and those described below were from New England Biolabs (NEB, Ipswich, MA) unless otherwise specified. The 100 μl end-repair mixture was incubated at 20°C for 30 min, purified by a PCR purification kit (Cat # 28104, Qiagen) and eluted with 32 μl of elution buffer (EB). (3) To A-tail, all 32 μl of end-repaired DNA was mixed with 5 μl of 10 × Buffer (NEB buffer 2), 10 μl of 1 mM dATP and 3 μl of Klenow (exo-). The 50 μl mixture was incubated at 37°C for 30 min before DNA was purified with a MinElute PCR purification kit (Cat # 28004, Qiagen). Purified DNA was eluted with 12.5 μl of 70°C EB and obtained with 10 μl of EB. (4) For adaptor ligation, 10 μl of A-tailed DNA was mixed with 10 μl of PE-adaptor (Illumina), 25 μl of 2x Rapid ligase buffer and 5 μl of Rapid Ligase. The ligation mixture was incubated at room temperature (RT) or 20°C for 15 min. (5) To purify adaptor- ligated DNA, 50 μl of ligation mixture from step (4) was mixed with 200 μl of NT buffer from NucleoSpin Extract II kit (cat# 636972, Clontech, Mountain View, CA) and loaded into a NucleoSpin column. The column was centrifuged at 14,000 g in a desktop centrifuge for 1 min, washed once with 600 μl of wash buffer (NT3 from Clontech), and centrifuged again for 2 min to dry completely. DNA was eluted in 50 μl elution buffer included in the kit. (6) To obtain an amplified library, ten PCRs of 25 μl each were set up, each including 13.25 μl of H_2_O, 5 μl of 5 × Phusion HF buffer, 0.5 μl of a dNTP mix containing 10 mM of each dNTP, 0.5 μl of Illumina PE primer #1, 0.5 μl of Illumina PE primer #2, 0.25 μl of Hotstart Phusion polymerase, and 5 μl of the DNA from step (5). The PCR program used was: 98°C 1 minute; 6 cycles of 98°C for 20 seconds, 65°C for 30 seconds, 72 °C for 30 seconds; and 72 °C for 5 min. To purify the PCR product, 250 μl PCR mixture (from the ten PCR reactions) was mixed with 500 μl NT buffer from a NucleoSpin Extract II kit and purified as described in step (5). Library DNA was eluted with 70°C elution buffer and the DNA concentration was estimated by absorption at 260 nm.

### Exome DNA Capture

Human exome capture was performed following a protocol from Agilent's SureSelect Paired-End Version 2.0 Human Exome Kit (Agilent, Santa Clara, CA) with the following modifications. (1) A hybridization mixture was prepared containing 25 μl of SureSelect Hyb # 1, 1 μl of SureSelect Hyb # 2, 10 μl of SureSelect Hyb # 3, and 13 μl of SureSelect Hyb # 4. (2) 3.4 μl (0.5 μg) of the PE-library DNA described above, 2.5 μl of SureSelect Block #1, 2.5 μl of SureSelect Block #2 and 0.6 μl of Block #3; was loaded into one well in a 384-well Diamond PCR plate (cat# AB-1111, Thermo-Scientific, Lafayette, CO), sealed with microAmp clear adhesive film (cat# 4306311; ABI, Carlsbad, CA) and placed in GeneAmp PCR system 9700 thermocycler (Life Sciences Inc., Carlsbad CA) for 5 minutes at 95°C, then held at 65°C (with the heated lid on). (3) 25-30 μl of hybridization buffer from step (1) was heated for at least 5 minutes at 65°C in another sealed plate with heated lid on. (4) 5 μl of SureSelect Oligo Capture Library, 1 μl of nuclease-free water, and 1 μl of diluted RNase Block (prepared by diluting RNase Block 1: 1 with nuclease-free water) were mixed and heated at 65°C for 2 minutes in another sealed 384-well plate. (5) While keeping all reactions at 65°C, 13 μl of Hybridization Buffer from Step (3) was added to the 7 μl of the SureSelect Capture Library Mix from Step (4) and then the entire contents (9 μl) of the library from Step (2). The mixture was slowly pipetted up and down 8 to 10 times. (6) The 384-well plate was sealed tightly and the hybridization mixture was incubated for 24 hours at 65°C with a heated lid.

After hybridization, five steps were performed to recover and amplify captured DNA library: (1) Magnetic beads for recovering captured DNA: 50 μl of Dynal MyOne Streptavidin C1 magnetic beads (Cat # 650.02, Invitrogen Dynal, AS Oslo, Norway) was placed in a 1.5 ml microfuge tube and vigorously resuspended on a vortex mixer. Beads were washed three times by adding 200 μl of SureSelect Binding buffer, mixing on a vortex for five seconds and then removing the supernatant after placing the tubes in a Dynal magnetic separator. After the third wash, beads were resuspended in 200 μl of SureSelect Binding buffer. (2) To bind captured DNA, the entire hybridization mixture described above (29 μl) was transferred directly from the thermocycler to the bead solution and mixed gently; the hybridization mix/bead solution was incubated in an Eppendorf thermomixer at 850 rpm for 30 minutes at room temperature. (3) To wash the beads, the supernatant was removed from beads after applying a Dynal magnetic separator and the beads were resuspended in 500 μl SureSelect Wash Buffer #1 by mixing on vortex mixer for 5 seconds, then incubated for 15 minutes at room temperature. Wash Buffer #1 was then removed from beads after magnetic separation. The beads were further washed three times, each with 500 μl pre-warmed SureSelect Wash Buffer #2 after incubation at 65°C for 10 minutes. After the final wash, SureSelect Wash Buffer #2 was completely removed. (4) To elute captured DNA, the beads were suspended in 50 μl SureSelect Elution Buffer, vortex-mixed and incubated for 10 minutes at room temperature. The supernatant was removed after magnetic separation, collected in a new 1.5 ml microcentrifuge tube, and mixed with 50 μl of SureSelect Neutralization Buffer. DNA was purified with a Qiagen MinElute column and eluted in 17 μl of 70°C EB to obtain 15 μl of captured DNA library. (5) The captured DNA library was amplified in the following way: 15 PCR reactions each containing 9.5 μl of H_2_O, 3 μl of 5 × Phusion HF buffer, 0.3 μl of 10 mM dNTP, 0.75 μl of DMSO, 0.15 μl of Illumina PE primer #1, 0.15μl of Illumina PE primer #2, 0.15 μl of Hotstart Phusion polymerase, and 1 μl of captured exome library were set up. The PCR program used was: 98°C for 30 seconds; 14 cycles of 98°C for 10 seconds, 65°C for 30 seconds, 72°C for 30 seconds; and 72°C for 5 min. To purify PCR products, 225μl PCR mixture (from 15 PCR reactions) was mixed with 450 μl NT buffer from NucleoSpin Extract II kit and purified as described above. The final library DNA was eluted with 30 μl of 70°C elution buffer and DNA concentration was estimated by OD260 measurement.

### Somatic Mutation Identification by Massively Parallel Sequencing

Captured DNA libraries were sequenced with the Illumina GAIIx Genome Analyzer, yielding 150 (2 × 75) base pairs from the final library fragments. Sequencing reads were analyzed and aligned to human genome hg18 with the Eland algorithm in CASAVA 1.6 software (Illumina). A mismatched base was identified as a mutation only when (i) it was identified by more than five distinct reads; (ii) the number of distinct reads containing a particular mismatched base was at least 10% of the total distinct reads; and (iii) it was not present in >0.5% of the reads in the matched normal sample. “Distinct reads” were defined as reads that had different sequences at either the 5' or 3' end of the sequenced fragment, thereby indicating that they originated from different template molecules. SNP search databases included the NCBI's database (http://www.ncbi.nlm.nih.gov/projects/SNP/).

### Evaluation of Genes in Additional Tumors and Matched Normal Controls.

For select genes mutated in more than one discovery sample, the coding region was sequenced in additional specimens when available. PCR amplification and Sanger sequencing were performed following protocols described previously[23].

## Supplementary Tables


